# Trust and distrust in information systems at the workplace

**DOI:** 10.7717/peerj.5483

**Published:** 2018-09-12

**Authors:** Meinald T. Thielsch, Sarah M. Meeßen, Guido Hertel

**Affiliations:** Department of Psychology, University of Münster, Münster, Germany

**Keywords:** Work, Information systems, Information technologies, Trust, Distrust, Performance, Well-being

## Abstract

Digitalization of work processes is advancing, and this is increasingly supported by complex information systems (IS). However, whether such systems are used by employees largely depends on users’ trust in these IS. Because there are few systematic studies on this topic, this research provides an initial exploration and validation of preconditions for trust in work-related IS. In Study 1, *N* = 30 professionals were asked to describe occupational incidents in which they had highly trusted or distrusted an IS. Content analysis of 111 critical incidents described in the in-depth interviews led to 12 predictors of trust and distrust in IS, which partly correspond to the structure of the established IS success model (Delone & McLean, 2003) but also exceed this structure. The resulting integrative model of trust in IS at work was validated in Study 2 using an online questionnaire with *N* = 179 professionals. Based on regression analyses, reliability (system quality) and credibility (information quality) of IS were identified as the most important predictors for both trust and distrust in IS at work. Contrasting analyses revealed diverging qualities of trust and distrust in IS : whereas well-being and performance were rated higher in trust events, experienced strain was rated higher in distrust events. Together, this study offers a first comprehensive model of trust in IS at work based on systematic empirical research. In addition to implications for theory advancement, we suggest practical implications for how to support trust and to avoid distrust in IS at work.

## Introduction

Today, work processes are increasingly characterized by high complexity, multitasking, and time pressure. Information management is essential in many businesses, with smart and connected products generating a multitude of new data (Porter & Heppelmann, 2014). As a growing number of businesses advance in the digitalization of workflows, the processing of information is more and more supported by complex computer-based information systems (IS). IS are combinations of hardware, software, and network services build to collect, process, organize, store, and disseminate information. Such systems support analysis, control, coordination, visualization, and decision-making in organizations. Typical examples are enterprise resource planning or customer relationship management tools.

But how are users adapting and dealing with such IS? From a business perspective, employees are usually expected to rely on IS in their work, but they often do not (e.g.,  Ash, Berg & Coiera, 2004; Butler & Gray, 2006), resulting in impairments and damages to the user, the client, and even the organization. For end users to accept and promote an IS in an organization, they must first trust it (e.g., Li, Hess & Valacich, 2008; McKnight et al., 2011; Silic, Barlow & Back, 2018; Thatcher et al., 2011). If employees trust in an IS, they will probably use it; if they distrust an IS, they will probably try to find ways to avoid it. While user’s trust in IS thus seems critical, the determinants of trust in IS at work have not been systematically investigated yet. Moreover, established models of IS use (e.g., Delone & McLean, 2003) do not include trust.

In the current work, we empirically explored predictors of employees’ trust and distrust in IS at work. Given the pioneering character of this research, we used a qualitative approach in Study 1, collecting and analyzing critical incidents of trust and distrust in IS at the workplace based on a structured interview technique (Flanagan, 1954). The findings of this qualitative step were categorized and compared with an established theoretical framework of IS use (Delone & McLean, 2003). The resulting integrated model of trust and distrust in IS at work was then validated in Study 2 using quantitative data based on a sample of 179 working professionals.

This research provides the following contributions to the literature: according to our knowledge, this is the first systematic and empirical exploration of key predictors for users’ trust and distrust in IS at the workplace. The resulting comprehensive model should support a more thorough understanding of psychological preconditions of IS adoption and use at work. Furthermore, we systematically compared predictors of trust and distrust in IS at work, considering the recent more general discussion of diverging mindsets for trust and distrust (Gefen, Benbasat & Pavlou, 2008; Seckler et al., 2015). Finally, the current research offers specific suggestions for software design and practical interventions in order to increase trust (and/or avoid distrust) in IT systems, which will thus help encourage users to rely on IS at work.

## Trust in Information Systems

In the near future, computer technology may affect nearly every occupation (Hertel et al., 2017; Mitchell & Brynjolfsson, 2017). While technology is constantly advancing and chip performance is still accelerating (Waldrop, 2016), the amount of available data is rapidly growing as well. In particular, smart and interconnected products generate a multitude of new data, reshaping whole business processes and leading to a so-called “Internet of things” (Porter & Heppelmann, 2014). In light of these developments, a growing number of IS gather, organize, analyze, and display task-related information. Modern IS provide decision aids based on available inputs and programmed routines. Even more, algorithms based on machine learning examine patterns in available data and autonomously develop possible solutions for given tasks (Avolio et al., 2014; Wenzel & Van Quaquebeke, 2018).

In general, information technologies are built to support users at the workplace, for example, by reducing communication costs and effort or saving employees’ cognitive resources for other tasks (Aral, Brynjolfsson & Van Alstyne, 2012). Yet, the adoption and diffusion of IT depends on users’ trust in the given systems (Silic, Barlow & Back, 2018; Thatcher et al., 2011). Trust can be defined as the willingness to depend on and be vulnerable to an IS in uncertain and risky environments (Gefen, Benbasat & Pavlou, 2008; Mayer, Davis & Schoorman, 1995; Meeßen, Thielsch & Hertel, in press; Wang & Emurian, 2005). Trust relations involve two parties: the trustor (in our research, the user) and the party to be trusted, the trustee (in our research, the IS). Moreover, trust is highly subjective, is affected by individual differences as well as situational factors, and leads people to act in certain ways (Wang & Emurian, 2005). At the workplace, trust predicts commitment, risk taking, and (the lack of) counterproductive work behaviors. The relationship between trust and job performance is at least as strong as relationships of performance with other attitudes such as job satisfaction (Colquitt, Scott & LePine, 2007). Especially in virtual teamwork, trust is a crucial prerequisite for positive team-related attitudes, information sharing, and high team performance (Breuer, Hüffmeier & Hertel, 2016; Jarvenpaa, Cantu & Lim, 2017). Even more, Hoff & Bashir (2015) stated that users’ trust is key to improving safety and productivity with respect to automated systems.

So far, existing research on trust in technology in the field of human–computer interaction (HCI) has mostly focused on e-commerce systems and perceptions of Web services (Beldad, De Jong & Steehouder, 2010). Major steps have been made in understanding how people develop trust in such systems and specific software products (e.g.,  Li, Hess & Valacich, 2008; Silic, Barlow & Back, 2018). However, e-commerce and Web services are usually approached voluntarily, with end users acting in the role of customers (e.g.,  Beldad, De Jong & Steehouder, 2010). In contrast, in the current research we focused on users in the role of employees accomplishing occupational tasks according to organizational demands. In this context, employees are perceived as being responsible for fulfilling their tasks and have to find optimal solutions to expected and unexpected challenges. Furthermore, work processes can be highly repetitive, demanding similar tasks in various situations. In such modern work environments, supporting IS are used frequently, and sometimes even become employees’ daily companions. Thus, compared to e-commerce systems and Web services, IS at work relate to different tasks in scenarios characterized by high complexity, potential uncertainty, and high individual responsibilities (Hou, 2012; Hoff & Bashir, 2015).

Obviously, workers only trust IS that fulfill work requirements and run without major failures or errors. But even if the technical implementation of an IS is optimal, users might distrust the IS due to perceived low quality of data (see Sillence et al., 2007). Data may be perceived as low quality for various reasons, such as because of erroneous programming or flawed algorithms within the IS, input or operating errors by other users, or a lack of users’ operating skills. In work settings, users’ distrust in an IS might be followed by a neglect of relevant data or unnecessary workarounds, which not only cost money but can also lead to major failures. On the other hand, major fiascos, such as the loss of NASA’s $125 million Mars Climate Orbiter in 1998, due to a simple conversion error from English units to metric units (Sauser, Reilly & Shenhar, 2009), illustrate that not every IS should be blindly trusted. Different cognitive processes might be relevant when users develop trust or distrust. As a result, current research treats trust and distrust separately and as being located on different dimensions (Gefen, Benbasat & Pavlou, 2008; Seckler et al., 2015).

The present paper provides a systematic and empirical exploration of key predictors for trust and distrust in IS at the workplace. In doing so, we work towards a comprehensive model leading to a better understanding of psychological preconditions of IS adoption, diffusion, and use at the workplace. In light of the scarcity of specific research on employees’ trust in IS, we started with an explorative study using the critical incident technique (Flanagan, 1954). As one major advantage, collecting critical incidents allows one to investigate true trust and distrust incidents instead of users’ mere assumptions and subjective theories about trustworthy IS. Moreover, a qualitative approach can identify various relevant predictors for trust in IS in one single study, as the in-depth interviews focus on very different work-related IS.

## Study 1

Study 1 explored critical factors for trust and distrust in IS at the workplace by collecting critical incidents of employees’ trust and distrust in IS at work via in-depth interviews (based on Flanagan, 1954).

### Method

#### Participants and systems used

We interviewed 30 professionals (16 male, 14 female) who used IS at work that provide decision-relevant information. All participants worked in different organizations from various types of businesses. The interviewees’ mean age was 32.03 years (*SD*_*age*_ = 9.80; range: 23–63). The interviewees referred to 37 different IS involved in administering data related to products (12), customers (11), businesses (five), patients (five), or human resources (four). On average, the interviewees used the respective IS for 2.87 years (*SD* = 2.92; *n* = 30) and 4.05 hours per day (*SD* = 3.06; *n* = 37).

#### Procedure

Participants were recruited through personal contacts without monetary incentives or compensation. The interviews were conducted face-to-face (11) or via telephone (19). At the beginning of each interview, interviewees were informed about the aims and procedure of the interview, that participation was voluntary, and that all data would be anonymized. Moreover, a consent to recording the interview was collected. Next, participants were asked to remember a situation in which they trusted an IS, and to describe all circumstances of that incident:

“Please remember a situation, in which you trusted an information system. In this situation, you should have relied heavily on the information system and you should have carried out a concrete action with the information system. Please describe all of the circumstances, and above all, please describe which prevailing conditions caused your trust in this situation.”

In case the participants did not provide sufficient details about the predictors for trust, their feelings, the situation’s consequences and the persons involved, these details were further obtained using open questions. After finishing the description of the first critical incident, further critical incidents for trust were inquired until the participants noted that they could not remember further situations.

Next, participants were asked to remember an incident in which they had distrusted an IS. Again, details were requested if necessary. This procedure was repeated for critical incidents of distrust until participants noted that they could not remember further situations. We deliberately did not restrict the time delay of the remembered trust and distrust incidents in order to capture also influencing factors that occur only rarely. At the end of the interviews, general information about the system and the participants’ use was collected (see Appendix A for the complete interview guide). The ethics committee of the FB07, the Faculty of Psychology and Sports Science of the University of Münster, granted ethical approval for this study (Ethical Application Ref: 2016-04-GH).

### Results

#### Content analysis

The interviews revealed 111 critical incidents, 57 for trust and 54 for distrust. The critical incidents for trust comprised 32 (56%) situations of data retrieval, 16 (28%) of general use, six (11%) of data input and management, and three (5%) of IS implementation and support. The critical incidents for distrust comprised 34 (63%) situations of data retrieval, 13 (24%) of data input and management, and seven (13%) of automated processes. Each critical incident was analyzed following Mayring (2000): first, for each critical incident, we extracted information from the interviewees’ statements related to the following units of analysis: predictors for trust or distrust, the incidents’ consequences, and the interviewees’ feelings associated with the incident. Second, predictors, consequences, and associated feelings were clustered to develop a system of categories. Third, the resulting predictors for trust or distrust were categorized and compared with the more general framework developed by DeLone & McLean (1992), Delone & McLean (2003) (theory-based embedding).

In this third step, we compared our findings to the *IS success model* of DeLone & McLean (1992), which was extended to its current form in 2003 (Delone & McLean, 2003) and is perhaps the most established theoretical model that describes users’ more general interactions with IS. In their model, the authors conceptualized the relationship between three main dimensions of information systems: (1) *information quality*, referring to the content of an IS and its completeness, comprehensibility, personalization, relevance, and security; (2) *system quality,* describing the technical quality of a system in terms of its adaptability, availability, reliability, response time, and usability; and (3) *service quality* (added to the model in 2003)*,* referring to general perceptions of assurance, empathy, and responsiveness of a service provider offering support to end users. Those three aspects influence usage intentions, actual system use, and user satisfaction, leading to individual and organizational consequences. In addition, Delone & McLean (2003) stress the importance of context variables (e.g., organization, employer support). Somewhat surprisingly, trust is not included in this model, making it difficult to explain trust in IS using this model (or similar existing approaches). Nevertheless, we considered the IS success model as a suitable, more general framework for our research and followed the recommendation of Delone & McLean (2003) to adapt their model to the objective and context of an investigation.

Two independent observers coded the data. Inter-rater agreement was calculated using Cohens *κ* (Cohen, 1960). The resulting *κ* = .83 for trust situations and *κ* = .91 for distrust situations indicate excellent intercoder reliability (see Table 1 for descriptions of the predictors and statement examples for trust and distrust mentioned in the interviews).

**Table 1:** Predictors for trust or distrust.

Factor	Description	Example of statements
**User**		
Trust in Technology^*^	Trust in electronic data or in technology in general.	“As data transfer is electronic I trust in it.” “A machine normally makes no errors.”
Experience & Skills^*^	Experience and skills dealing with the system.	“Over time, one gets a feeling if [information of the system] is correct or not.”
**System Quality**		
Reliability	Past experiences regarding dependability, lack and correctness of data, technical verification, and distribution of the system.	“The error already occurred too often.” “[The system] worked that reliable that I did not worry about not being able to provide the proper documents.”
Implemented controls^*^	Traceability, automation, backups, data checking, and additional IS.	“If I want to change customer information and enter the name of a street, I can be completely sure that it [the address] is automatically established and that it is definitely correct.”
Ease of Use	Usability and visualization.	“It is easy to use. […] If you did that [operation] a few times you are familiar with the system, because you can’t do much wrong, because the system is designed simple.”
**Information Quality**		
Content	Available data.	“The system lives on […] the quality of [data] entries.”
Security	Use of passwords, personal accounts, and internal networks.	“The system is only available via intranet. One cannot log in from outside.”
**Service Quality**		
Support	Maintenance, contact person in case of problems.	“Given that I have a contact person if a problem occurs, I trust even more in the task that I am executing.”
**Context**		
Participation & Transparency^*^	Background information about the IS, involvement at IS implementation and troubleshooting, autonomous data entry.	“In informal situations, during lunch break, I got information from my superior which made me aware that the implementation process was done seriously.”
Inevitability of Use^*^	Lack of alternatives.	“Without relying on the system, I would not be able to accomplish my work.”
Accountability	Obligation towards superiors, colleagues or clients.	“I feel sorry and embarrassed to give our clients false guarantees.”
Persons involved^*^	Attitude, ability, controls, and handling.	“I trust the system in itself. The problem are always the people in front of it.” “[The person responsible for data entry] is not always very reliable.”

**Notes.**

Factors mentioned in the interviews that extend the model by Delone & McLean (2003) are highlighted with asterisks.

#### Predictors and consequences

In sum, 134 statements regarding predictors for trust and 87 statements regarding predictors for distrust were provided by the participants. Table 2 shows the number of statements on different predictors for trust and distrust categorized as related to the user, system quality, information quality, service quality, context or persons involved. Mentioned most often were system quality (especially *Reliability*), context factors (mostly *Participation and Transparency* issues), and characteristics of *Persons involved*.

**Table 2:** Predictors for trust and/or distrust: absolute and relative number of statements.

Factor	Trust (%)	Distrust (%)
**User**	**16 (12)**	**5 (6)**
Trust in technology^*^	12 (9)	–
(insufficient) Experience & Skills^*^	4 (3)	5 (6)
**System Quality**	**58 (44)**	**50 (57)**
(Un)Reliability	33 (25)	45 (52)
(insufficient) Implemented controls^*^	20 (15)	2 (2)
(insufficient) Ease of use	5 (4)	3 (3)
**Information Quality**	**12 (9)**	**–**
Security	7 (5)	–
Content	5 (4)	–
**Service Quality**	**6 (4)**	**–**
Support	6 (4)	–
**Context**	**26 (19)**	**7 (8)**
(insufficient) Participation & Transparency^*^	23 (17)	5 (6)
Inevitability of use^*^	3 (2)	–
Accountability	–	2 (2)
Persons involved^*^	**16 (12)**	**25 (29)**

**Notes.**

Percentages are shares in total statements for trust and distrust respectively.

Factors mentioned in the interviews that extend the model by Delone & McLean (2003) are highlighted with asterisks.

Furthermore, 51 interviewee statements referred to the consequences of critical incidents for trust in IS, including that trusting the IS improved job performance (21), allowed them to execute follow-up actions (12), resulted in extending IS use (nine), facilitated their work (six), had a positive impact on stakeholders (two), and increased their trust in organizational support (one). Feelings mentioned to be associated with critical incidents for trust in IS included feelings of security (20), a general positive emotional state (11), feelings of carefreeness (three), confirmation (two), absolute trust (one), and relief from responsibility (one).

Regarding the consequences of critical incidents for distrust, participants gave 101 statements. In these statements, employees mentioned that distrusting the IS reduced job performance (36), caused employees to find alternative ways to execute the task (30), resulted in continuous distrust while using it (eight), negatively impacted stakeholders (eight), led employees to implement additional steps or functions (seven), portrayed a negative external image (six), led employees to distrust their colleagues (three), led to less system exploration (two), and prevented the communication of data (one). Feelings associated with those critical incidents for distrust were insecurity (15), annoyance (seven), helplessness (nine), shame (three), demotivation (two), and dissatisfaction (two).

### Discussion

Study 1 identified several predictors for trust and distrust in IS. Whereas some predictors correspond to facets of the IS success model (DeLone & McLean, 1992; Delone & McLean, 2003), others extended this model as additional predictors of trust (see Table 2). Specifically, as a result of Study 1, we added user variables, the additional system quality *Implemented controls*, additional context variables such as *Participation and Transparency* and *Inevitability of use*, as well as characteristics of the *Persons involved*. The relevance of *User* variables can be supported by existing HCI literature, as this aspect is enclosed in other models of user experience (e.g., Thüring & Mahlke, 2007) or has proved to be important for digitalized work environments such as virtual teamwork (e.g., Schulze & Krumm, 2017). The role of *Context* factors for users’ trust was also stressed by authors investigating e-commerce environments (e.g., Cheung & Lee, 2006; Shankar, Urban & Sultan, 2002). Yet, e-commerce environments are different from the workplace, and e-commerce environments allow users to access for several different service providers, whereas at the workplace employees usually have no choice in the IS they must use. Employees’ only options are to use the given IS or find a workaround. Additional aspects that are more relevant at the workplace than in e-commerce are the amount of control users have over the technical system and the persons involved. Both factors were stated frequently as crucial for developing trust in work-related IS. In addition, with respect to service quality, statements about *Support* included help from service providers as well as aspects of support within the organization.

Surprisingly, participants of Study 1 did not mention the perceived credibility of a given IS, even though this construct has been stressed often in IS research (e.g., De Wulf et al., 2006; Metzger, 2007; Wathen & Burkell, 2002). Credibility is described as the believability of information and/or its source (e.g., Fogg & Tseng, 1999; Fogg et al., 2001), as well as a receiver-based judgement with the two primary dimensions of expertise and trustworthiness (see Metzger, 2007). It is possible that in the present interview study, participants relied on other aspects as indicators for credibility, for instance the perceived quality of information (e.g., Appelman & Sundar, 2016; Metzger & Flanagin, 2013), and thus did not explicitly mention credibility itself. However, one limitation of Study 1 as a qualitative approach is that not all potentially relevant aspects were covered. Thus, the predictors revealed in Study 1 have to be validated and extended by further theoretically derived predictors in a follow-up study using quantitative data.

In Study 2, the information quality dimension was amended by credibility. Moreover, to diversify aspects mentioned in the interviews, additional information regarding content aspects (such as *Amount*, *Clarity*, and *Relevance* of information) were included in Study 2. With respect to consequences of trust and distrust, Study 1 revealed several aspects in addition to the impact and performance indicators named by Delone & McLean (2003). Thus, individually experienced outcomes such as perceived stress or emotional well-being were further investigated in Study 2. Finally, in Study 1 more predictors for trust were stated than for distrust. Additionally, only *Reliability* of the IS and *Persons involved* were named frequently as predictors of distrust. Thus, in Study 2, potential differences between predictors of trust and predictors of distrust in IS at the workplace were analyzed in detail.

## Study 2

The objective of Study 2 was intended to validate the critical predictors for trust in IS at the workplace that were revealed in Study 1 and structured along the IS success model (Delone & McLean, 2003). In addition, we added further potential predictors theoretically derived from the DeLone and McLean model. Study 2 also differentiated between critical factors for trust and distrust in IS.

### Method

We conducted an online survey recruiting participants via the non-commercial German online access panel PsyWeb (https://psyweb.uni-muenster.de/). A between-subject design was used with situational trust vs. situational distrust as the dependent variable. Participants were randomly assigned to a condition in which they were asked to remember a situation of trust or a situation of distrust in an IS that they use at work.

#### Participants and systems used

In total, 545 persons started the survey (*n* = 282 in the trust and *n* = 263 in the distrust condition). Of them, 242 participants completed the study and agreed to have their data analyzed. The study’s focus was on IS that are used at the workplace. Thus, we excluded 63 datasets of people who mentioned software that did not represent an IS that provides decision-relevant information at work (e.g., email services, private online banking tools, Office suites, etc.). The initial and final sample did not differ significantly regarding age or gender. The final sample included 179 participants: *n* = 109 in the trust and *n* = 70 in the distrust condition. In the trust (distrust) condition 75% (79%) of the participants used the IS several times per week or on a daily basis (see Table 3 for further details). All participants completed the study anonymously, voluntarily, and without any compensation.

**Table 3:** Information on participants and administered data type for the trust and the distrust condition.

Information	Trust condition (*n* = 109)	Distrust condition (*n* = 70)
Age	M_age_ = 48.74 years (SD_age_ = 9.56; range: 26–63)	M_age_ = 47.86 years (SD_age_ = 8.88; range: 25–64)
Sex	59 female, 50 male	37 female, 33 male
Period of use	*M* = 7.04 years (SD = 5.44; range: 0.25–30)	*M* = 6.83 years (SD = 5.85; range: 0.5–32)
Data type		
Customer data	20%	39%
Product data	19%	19%
Patient data	15%	6%
Business data	12%	14%
HR data	6%	4%
Programming data	6%	3%
Others	22%	16%

#### Procedure

The Web-based survey was offered in German with EFS Survey 10.8 (provided by the Questback GmbH) and explicitly addressed persons who used IS at work that provide decision-relevant information. Panel members received an invitation email with a link to the survey, accessible for four weeks. Participants were informed about involved researchers, anonymity, voluntariness, and duration of the study. After providing demographic information and answering items on user-related factors, participants were asked to remember a situation in which they trusted an IS or a situation in which they distrusted an IS (using the same critical incident question as in Study 1). Afterwards, information about the IS and the situation were assessed, followed by the measure of situational trust or situational distrust. After that, the survey asked about measures of factors concerning system quality, information quality and context as well as consequences such as well-being, stress, performance, and further use of the system, all relating to the remembered situation. At the end, participants had the option to exclude their data from subsequent analysis and comment on the study, and were then thanked. On average, participants needed 12.65 minutes to complete the survey.

#### Measures

Study 2 incorporates several established and validated measures from HCI and IS research. Yet, with respect to trust, research so far has not been able to clearly differentiate between trust and trustworthiness—most existing measures are focused only on trustworthiness (see Gefen, Benbasat & Pavlou, 2008). Consequently, we developed three items to measure situational trust and distrust.

User-related measures included the German *Technology competence* scale (Neyer, Felber & Gebhardt, 2012) and the adapted *Trusting stance in general technology* scale (McKnight et al., 2011). The *Regular frequency of use* was directly assessed by one item.

As system quality measures, we adapted the *Reliability* scale from McKnight and colleagues (McKnight, Choudhury & Kacmar, 2002; McKnight et al., 2011), the *Ease of use* scale from Gefen & Keil (1998), and the *Response time* scale from Palmer (2002). In order to assess *Implemented controls*, we created three items on the basis of the integrity and security scales of Rivard and colleagues (1997).

As information quality measures, we adapted scales measuring appropriate *Amount*, *Security*, and *Relevance* from the AIMQ (Lee et al., 2002) as well as the *Clarity*, *Credibility*, and *Informativeness* scales from the Web-CLIC (Thielsch & Hirschfeld, in press).

Given that the predictor *Support*, which was revealed in Study 1, concerned both support staff and a general supportive climate, we separated this factor. The existence of support as a service quality factor was measured with three items developed for this study.

As a context factor, *Perceived organizational support* was measured with a scale from Eisenberger and colleagues (2001). As further context factor measures, we adapted measures from Baroudi & Orlikowski (1988) to assess *Participation* and *Transparency*. The *Communication of errors* was measured with a scale for safety communication (Cigularov, Chen & Rosecrance, 2010). *Obligation to use* the system was measured with two items adapted from Moore & Benbasat (1991).

Predictors revealed in Study 1 concerning involved persons were measured with two scales from the VIST model considering *Abilities* and *Attitudes* of involved persons (Hertel, Konradt & Orlikowski, 2004) as well as one scale considering *Accountability* to involved persons (Frink & Ferris, 1998).

For consequences, we used the smiley rating scale from Jäger (2004) to assess *Well-being* and adopted three items from the *Stress in general* scale (Stanton et al., 2001). For measuring *User performance*, we adopted the scale from Etezadi-Amoli & Farhoomand (1996). Post-situational *Frequency of use* and *Satisfaction with use* were directly assessed with one item each.

Most items were measured with a seven-point Likert scale. Further explorative variables were measured that are not considered in this study (see Appendix B) for the full item list).

#### Reported incidents

In the trust (distrust) condition, 15% (4%) of the remembered incidents happened on the same day as the survey, 36% (11%) during the previous week, 28% (34%) during the previous month, 15% (31%) during the previous year, and 7% (19%) more than one year ago. 28% (none) of the remembered situations occurred on a daily basis, 25% (6%) several times per week, 9% (6%) once per week, 11% (19%) several times per month, 4% (11%) once per month, 16% (30%) several times per year, 2% (11%) once per year, and 6% (17%) once. In sum, 39% (30%) of the incidents referred to data retrieval, 39% (29%) data input and management, 12% (17%) automated processes, 6% (10%) data security, and 6% (13%) system implementation and support.

### Results

The effects of the considered predictors on situational trust were examined using regression analyses. First, we regressed trust on the six main quality dimensions—i.e., user, system quality, information quality, service quality, context, and persons involved—using six separate regression analyses. As displayed in Table 4, the multiple regression on user factors revealed that none of the conserved factors had a significant effect on trust. Thus, individual characteristics seem to play a minor role for trust in IS at work, at least in the current study. In the multiple regression on the first quality dimension in the DeLone and McLean model, system quality, the only significant predictor of trust was *Reliability*. The multiple regression on the second quality dimension in the DeLone and McLean model, information quality, revealed *Credibility* as the only significant predictor of trust. In the multiple regression on the third quality dimension in the DeLone and McLean model, service quality, *Support* was a significant predictor of trust. Concerning possible relevant context factors, *Participation* was significantly associated with trust. With respect to the persons involved, which comprised an additional group of factors not considered in the DeLone and McLean model but deemed important in Study 1, *Ability* was significantly associated with trust.

**Table 4:** Multiple regression analyses predicting situational trust or situational distrust. Separately for factors concerning user, system quality, information quality, context, and persons involved (step 1) and commonly for significant predictors of step 1 (step 2).

	Trust			Distrust		
Predictor	*R*_*adj*_^2^	*B*	*β*	*R*_*adj*_^2^	*B*	*β*
Step 1						
User	.04			−.00		
Technology competence		.19	.16		.09	.07
Trust in technology		.13	.15		−.02	−.02
Regular frequency of use		.09	.10		−.17	−.20
System quality	.51^***^			.13^*^		
Reliability		.60^***^	.66^***^		−.42^**^	−.37^**^
Controls		.08	.07		−.04	−.03
Ease of use		.01	.01		−.11	−.11
Response time		.03	.05		.03	−.03
Information quality	.40^***^			.20^**^		
Amount		.04	.05		−.35^*^	−.35^*^
Relevance		.08	.05		−.31	−.23
Security		.07	.07		.13	.12
Informativeness		.07	.06		.16	.13
Credibility		.57^***^	.52^***^		−.44^*^	−.35*
Clarity		.09	.10		.31^*^	.26^*^
Service quality	.08^**^			−.01		
Support		.29^**^	.31^**^		−.10	−.10
Context	.22^***^			−.04		
Participation		.26^**^	.35^**^		−.23	−.21
Transparency		.05	.08		.02	.02
Error communication		.13	.09		.01	.01
Perceived organizational support		.14	.16		.05	.05
Obligation to use		.04	.04		−.02	−.02
Persons involved	.15^***^			.09^*^		
Ability		.30^**^	.36^**^		−.13	.−13
Attitude		.08	.09		−.29	.−29
Accountability		.15	.09		.11	.08
Step 2	.60^***^			.25^***^		
Reliability		.45^***^	.50^***^		−.30^*^	−.26
Credibility		.37^***^	.34^***^		−.35^*^	−.28
Support		.02	.02		–	–
Participation		.04	.06		–	–
Ability		.04	.04		–	–
Amount		–	–		−.24	−.24
Clarity		–	–		.29^*^	−.24^*^

**Notes.**

* *p* < .05.

** *p* < .01.

*** *p* < .001.

In a second step, we calculated an overall regression integrating all factors that were revealed as significant predictors in the preceding analyses. As displayed in Table 4, this overall analysis revealed *Reliability* and *Credibility* as strongest predictors of trust in IS at work. This supports the overall relevance of both factors and further justifies the integration of *Credibility* as an additional factor as discussed in Study 1.

In order to examine potential differences regarding critical factors for distrust in comparison to trust, we also analyzed the various potential predictors with respect to distrust as the dependent variable. Again, we applied a two-stage procedure. First, regression analyses were calculated separately for the main quality dimensions—i.e., user, system quality, information quality, service quality, context, and persons involved (see Table 4 for detailed results). Similar to the results for trust, user-related factors were not related to distrust in IS in Study 2. As for trust, *Reliability* was negatively associated with distrust (as the only factor of system quality), with higher *Reliability* being related to lower distrust. *Credibility* (an information quality factor) was also negatively related to distrust. Moreover, *Amount* and *Clarity* of information were related to distrust in IS. However, contrary to our expectations, *Clarity* was positively related to distrust, i.e., higher *Clarity* was accompanied by higher distrust in the regression even though distrust and *Clarity* were uncorrelated in bivariate analyses (*r* =  − .04, see Table 5). This pattern might indicate a suppressor effect in which the suppressor variable has zero or close to zero correlation with the criterion but is correlated with other predictor variables (Nathans, Oswald & Nimon, 2012). Indeed, *Clarity* was correlated with most other information quality factors in Study 2 (see Table 5). In multiple regression, a suppressor variable increases the predictive power of other predictor variables because it allows one to control for irrelevant variance (see Nathans, Oswald & Nimon, 2012). To clarify which variables were affected by the observed suppressor effect, we calculated additional multiple regressions adding information quality factors stepwise with *Clarity* as the last factor. These analyses revealed that *Credibility* of information, *Amount* of information, *Relevance*, and *Informativeness* of content only affected distrust significantly when considered separately. Adding *Clarity* as the last factor into a combined regression increased the effects of *Amount* of information from *β* =  − .24, *t*(64) =  − 1.75, *p* = .085 up to *β* =  − .35, *t*(63) =  − 2.42, *p* = .019, and of *Credibility* of information from *β* =  − .29, *t*(64) =  − 1.79, *p* = .078 up to *β* =  − .35, *t*(63) =  − 2.17, *p* = .034. Thus, even though *Clarity* was not related to distrust in the bivariate correlation, considering *Clarity* in the multiple prediction of distrust specified the effects of *Amount* and *Credibility* of information by controlling for irrelevant pieces of variance in the latter two factors. It seems that users’ distrust in IS is based on those parts of *Amount* and *Credibility* of information that are independent of *Clarity* of information. On the other hand, those parts of variance in *Clarity* that were unrelated to *Amount* and *Credibility* of information were even positively related to distrust. Thus, *Clarity* of information per se seems not to automatically foster trust in IS but might even be related to distrust when *Clarity* (as a more formal criteria) is not connected with sufficient and credible information.

**Table 5:** Intercorrelations for measures as a function of incidents of trust vs. distrust.

Measure	1	2	3	4	5	6	7	8	9	10	11	12	13	14	15	16	17	18	19	20	21	22	23	24	25	26	27	28
1 Technology competence		.08	.09	−.05	.08	.05	−.08	.07	.35^**^	.24^*^	.07	−.06	.11	.29^*^	.51^***^	.12	.11	.27^*^	−.08	.02	−.07	.15	−.03	−.14	−.14	−.10	.01	.05
2 Trust in technology	.04		.03	−.02	.07	.08	.04	−.03	.10	.12	−.01	.23	.05	−.08	−.12	.09	.05	.02	.04	−.01	.02	.03	.16	−.06	.02	−.06	−.16	−.02
3 Regular frequency of use	.17	.05		.29^*^	.21	.10	−.09	.21	.4^***^	.27^*^	.29^*^	.22	.06	.05	.03	.39^***^	.30^*^	−.01	.47^***^	.22	.13	.33^**^	−.01	.13	−.01	.07	.35^**^	−.19
4 Reliability	.07	.02	−.06		.40^***^	.29^*^	.22	.46^***^	.47^***^	.29^*^	.35^**^	.34^**^	.27^*^	.23	.06	.38^**^	.07	.20	.23	.39^**^	.35^**^	.17	.24^*^	−.20	.20	.34^**^	.60^***^	−.41***
5 Controls	.15	.07	.09	.50^***^		.29^*^	−.01	.27^*^	.22	.20	.16	.25^*^	.18	.26^*^	.05	.23	−.05	−.08	.12	.07	.02	.11	−.04	−.05	.00	.00	.15	−.21
6 Ease of use	−.05	−.09	.14	.32^***^	.32^***^		.29^*^	.33^**^	.28^*^	.06	.26^*^	.32^**^	.64^***^	.34^**^	.16	.21	−.01	.10	−.02	.13	.17	.12	.21	−.15	.18	.06	.25^*^	−.21
7 Response time	.08	.03	−.04	.45^***^	.29^**^	.35^***^		.30^*^	.17	.14	.16	.22	.49^***^	.30^*^	.03	.22	.11	.19	−.03	.20	.27^*^	.13	.07	−.09	.27^*^	.13	.28^*^	−.08
8 Amount	.01	−.12	−.01	.49^***^	.33^***^	.26^**^	.41^***^		.45^***^	.34^**^	.57^***^	.46^***^	.49^***^	.38^**^	.21	.25^*^	.20	.27^*^	.15	.43^***^	.29^*^	.16	.09	−.05	.22	−.08	.40^***^	−.37**
9 Relevance	.21^*^	−.14	.33^***^	.24^*^	.26^**^	.28^**^	.23^*^	.33^***^		.49^***^	.63^***^	.48^***^	.31^**^	.29^*^	.33^**^	.45^***^	.31^**^	.36^**^	.38^**^	.43^***^	.31^*^	.45^**^	.08	−.09	.15	.14	.43^***^	−.33**
10 Security	.15	−.12	.21^*^	.09	.14	−.14	.08	.12	.08		.33^**^	.37^**^	.21	.34^**^	.27^*^	.41^***^	.20	.24^*^	.31^**^	.42^***^	.42^***^	.15	−.05	.03	.17	.22	.34^**^	−.14
11 Informativeness	.13	−.03	.07	.50^***^	.32^***^	.25^**^	.33^***^	.40^***^	.58^***^	.09		.73^***^	.32^**^	.34^**^	.17	.27^*^	.19	.29^*^	.20	.46^***^	.36^**^	.23	.06	.11	.27^*^	.05	.24^*^	−.35**
12 Credibility	.19^*^	−.03	.04	.52^***^	.30^**^	.19^*^	.39^***^	.44^***^	.36^***^	.09	.62^***^		.36^**^	.20	−.04	.29^*^	.20	.23	.25^*^	.37^**^	.40^***^	.29^*^	.15	−.03	.22	.07	.06	−.39***
13 Clarity	−.04	−.01	.07	.43^***^	.42^***^	.62^***^	.47^***^	.39^***^	.46^***^	−.02	.5^***^	.33^***^		.39^***^	.15	.36^**^	.10	.33^**^	−.02	.28^*^	.24^*^	.13	.11	−.15	.29^*^	−.04	.32^**^	−.04
14 Participation	.35^***^	.00	.15	.54^***^	.41^***^	.09	.36^***^	.40^***^	.27^**^	.21^*^	.32^***^	.33^***^	.25^**^		.55^***^	.22	.04	.30^*^	.01	.26^*^	.27^*^	−.05	.06	.01	.11	−.10	.30^*^	−.18
15 Transparency	.34^***^	.01	.15	.38^***^	.40^***^	.18	.39^***^	.32^***^	.28^**^	.20^*^	.35^***^	.24^*^	.41^***^	.64^***^		.09	−.01	.27^*^	−.06	.01	−.01	−.14	.05	−.03	.12	−.03	.23	−.08
16 Support	.24^*^	.17	.08	.27^**^	.43^***^	.18	.36^***^	.28^**^	.37^***^	.15	.40^***^	.35^***^	.38^***^	.35^***^	.21^*^		.40^***^	.20	.20	.53^***^	.49^***^	.29^*^	.07	−.07	.26^*^	.00	.31^**^	−.10
17 Error communication	.18	.08	.28^**^	.08	.32^***^	.02	.20^*^	.18	.43^***^	.04	.26^**^	.18	.18	.27^**^	.19	.44^***^		.36^**^	.25^*^	.45^***^	.41^***^	.40^**^	.14	−.13	.09	−.13	.10	.01
18 Perceived organizational support	.15	.11	.08	.26^*^	.34^***^	.10	.16	.23^*^	.35^***^	.13	.44^***^	.23^*^	.26^**^	.27^**^	.27^**^	.32^***^	.35^***^		.06	.46^***^	.23	.17	.26^*^	−.19	.15	.09	.21	−.01
19 Obligation to use	−.09	.25^**^	.31^***^	−.17	−.04	−.01	−.15	−.12	.08	−.12	−.06	.08	−.02	−.08	−.09	−.05	−.05	−.07		.17	.13	.63^***^	−.11	.23	−.10	.10	.17	−.02
20 Ability^a^	.15	.16	−.02	.45^***^	.31^***^	.13	.35^***^	.34^***^	.23^*^	.03	.30^**^	.40^***^	.41^***^	.47^***^	.40^***^	.48^***^	.30^**^	.32^***^	−.02		.64^***^	.23	.33^**^	−.08	.33^**^	.11	.39^***^	−.24*
21 Attitude^a^	.04	.19	.11	.33^***^	.33^***^	.16	.35^***^	.20^*^	.32^**^	.04	.32^**^	.31^**^	.43^***^	.29^**^	.30^**^	.48^***^	.43^***^	.43^***^	.05	.65^***^		.26^*^	.27^*^	−.17	.24^*^	.19	.34^**^	−.30*
22 Accountability^a^	.15	.24^*^	.05	−.02	.03	−.03	.01	−.11	.15	−.01	.11	.00	−.05	−.01	−.05	.18	.27^**^	.14	.11	.16	.25^*^		.00	.00	−.02	−.02	.03	−.02
23 Well-being	.13	.01	.02	.57^***^	.38^***^	.38^***^	.41^***^	.39^***^	.34^***^	.06	.43^***^	.49^***^	.48^***^	.53^***^	.40^***^	.36^***^	.19^*^	.32^***^	−.18	.45^***^	.36^***^	.03		−.61	.36^**^	.10	.31^*^	−.22
24 Stress	−.29^**^	.03	−.02	−.33^***^	−.16	−.24^*^	−.27^**^	−.39^***^	−.24^*^	−.09	−.33^***^	−.49^***^	−.28^**^	−.34^***^	−.26^**^	−.28^**^	−.01	−.17	.04	−.30^**^	−.29^**^	−.16	−.57^***^		−.16	−.01	−.27^*^	.00
25 Performance	.15	.02	.00	.68^***^	.41^***^	.37^***^	.44^***^	.48^***^	.37^***^	.03	.57^***^	.51^***^	.51^***^	.51^***^	.35^***^	.45^***^	.20^*^	.36^***^	−.14	.48^***^	.45^***^	.14	.74^***^	−.54^***^		.01	.21	−.08
26 Post-situational frequency of use	.14	.14	.15	.39^***^	.20^*^	.21^*^	.26^**^	.21^*^	.25^**^	.01	.25^**^	.30^**^	.41^***^	.34^***^	.22^*^	.26^**^	.16	.20^*^	.11	.36^***^	.35^***^	.13	.43^***^	−.22^*^	.42^**^		.51^***^	−.34**
27 Post-situational satisfaction with use	.10	.06	.08	.60^***^	.41^***^	.40^***^	.39^***^	.46^***^	.38^***^	.02	.44^***^	.50^***^	.47^***^	.44^***^	.36^***^	.35^***^	.14	.29^**^	.11	.44^***^	.31^**^	.09	.71^***^	−.48^***^	.69^***^	.60^***^		−.35**
28 Situational trust /distrust	.18	.16	.13	.72^***^	.42^***^	.26^**^	.37^***^	.36^***^	.34^***^	.13	.49^***^	.63^***^	.34^***^	.46^***^	.36^***^	.31^**^	.26^**^	.30^**^	−.01	.44^***^	.34^***^	.06	.57^***^	−.36^***^	.58^***^	.31^**^	.49^***^	

**Notes.**

Intercorrelations for the distrust condition (*n* = 70) are presented above the diagonal, and intercorrelations for the trust condition (*n* = 109) are presented below the diagonal.

a Reduced *n* due to response category “does not apply”, minimum *n* = 95 in the trust condition and *n* = 63 in the distrust condition.

* *p* < .05.

** *p* < .01.

*** *p* < .001.

In the second step, upon integrating all factors that were revealed as significant predictors of distrust in the preceding analyses, we again found a positive effect of *Clarity* whereas *Credibility* of information was negatively associated with distrust. Also, *Reliability* revealed a significant negative relation with distrust in step 2 (see Table 4).

In sum, our analyses showed that *Reliability* and *Credibility* of information were the most important predictors for both trust and distrust in IS.

In order to further examine differences in the factors between the trust and distrust condition, we calculated *t*-tests for independent samples (see Table 6 for results). User-related factors did not differ between the trust and distrust conditions. This indicates that concerning technology competence, trust in technology, and frequency of use, participants who experienced trust in IS at the workplace did not differ systematically from participants who experienced distrust. This is in line with the weak correlations between trust or distrust with user factors (see Table 5) and the findings of the regression analyses.

**Table 6:** Factors in the trust and distrust condition: descriptives, internal consistency and contrasts.

	Trust (*n* = 109)				Distrust (*n* = 70)	
Factor	*M* (*SD*)	*α*	*t*-test	Cohen’s *d*	*M* (*SD*)	*α*
User						
Technology competence	6.18 (1.02)	.921	n.s.	0.089	6.09 (0.99)	.901
Trust in technology	4.55 (1.47)	.909	n.s.	−0.051	4.62 (1.18)	.910
Regular frequency of use	6.92 (1.33)	–	n.s.	0.033	6.87 (1.75)	–
System quality						
Reliability	4.89 (1.35)	.893	*t*(177) = 3.88^***^	0.594	4.10 (1.30)	.852
Controls	4.90 (1.14)	.517	*t*(177) = 3.32^**^	0.510	4.33 (1.08)	.405
Ease of use	4.24 (1.60)	.930	n.s.	0.039	4.18 (1.44)	.923
Response time	4.91 (1.72)	.988	*t*(177) = 2.38^*^	0.362	4.31 (1.56)	.964
Information quality						
Amount	4.80 (1.41)	.841	*t*(177) = 2.89^**^	0.448	4.16 (1.46)	.819
Relevance	5.97 (0.81)	.824	*t*(177) = 3.02^**^	0.456	5.55 (1.07)	.872
Security	5.66 (1.25)	.896	*t*(177) = 2.87^**^	0.438	5.08 (1.43)	.916
Informativeness	5.46 (0.99)	.880	*t*(177) = 3.69^***^	0.559	4.86 (1.19)	.861
Credibility	5.66 (1.13)	.941	*t*(177) = 4.57^***^	0.698	4.86 (1.17)	.916
Clarity	4.59 (1.28)	.790	n.s.	0.190	4.35 (1.23)	.744
Service quality						
Support	5.46 (1.29)	.849	*t*(177) = 2.66^**^	0.414	4.91 (1.39)	.838
Context						
Participation	3.80 (1.66)	.778	n.s.	0.246	3.42 (1.35)	.591
Transparency	4.08 (1.86)	.809	n.s.	0.142	3.83 (1.61)	.851
Error communication	5.91 (0.92)	.905	*t*(177) = 3.37^***^	0.513	5.40 (1.10)	.887
Perceived organizational support	4.70 (1.41)	.916	n.s.	0.209	4.40 (1.48)	.880
Obligation to use	6.06 (1.39)	.832	n.s.	−0.041	6.12 (1.60)	.950
Persons involved						
Ability	5.31 (1.48)^a^	.957	*t*(173) = 2.50^*^	0.390	4.73 (1.50)	.932
Attitude	5.20 (1.40)^b^	.894	n.s.	0.239	4.88 (1.44)	.875
Accountability	6.46 (0.76)^c^	.688	n.s.	0.228	6.25 (1.03)^d^	.905
Outcome						
Well-being	3.37 (0.96)	–	*t*(175.5) = 7.45^***^	1.060	2.46 (0.67)	–
Stress	3.46 (1.49)	.950	*t*(166.91) = − 3.51^***^	−0.518	4.18 (1.22)	.925
Performance	4.66 (1.47)	.914	*t*(177) = 5.88^***^	0.898	3.41 (1.26)	.884
Post-situational frequency of use	4.05 (0.85)	–	n.s.	0.212	3.87 (0.85)	–
Post-situational satisfaction with use	4.42 (1.47)	–	*t*(177) = 2.39^*^	0.367	3.91 (1.25)	–
Situational trust/distrust	5.47 (1.23)	.875	*t*(177) = 5.45^***^	0.828	4.37 (1.47)	.821

**Notes.**

*α* standardized coefficient alpha n.s. not significant

a *n* = 105.

b *n* = 103.

c *n* = 98.

d *n* = 63.

* *p* < .05.

** *p* < .01.

*** *p* < .001.

Most system quality factors (i.e., *Reliability, Controls, Response time*) were rated significantly higher in the trust condition than in the distrust condition. The same was found for information quality factors (*Amount* of information, *Relevance, Security, Informativeness,* and *Credibility*), as well as for the one investigated service quality factor (*Support*). The context factor *Error communication* as well as the person factor *Ability* were also rated higher in the trust condition. In sum, high quality IS were associated with trust—in distrust situations, evaluated IS received lower but mostly still medium ratings.

Among the outcome variables, *Performance, Well-being* and *Post-situational satisfaction* with IS use were significantly higher in the trust condition, whereas *Stress* was significantly lower in the trust condition. This is in line with the high correlation of those outcome variables with trust (see Table 5), whereas outcome variables such as post-situational satisfaction with use and frequency of use were significantly correlated with distrust. In addition, situational trust was rated significantly higher than situational distrust. Thus, in reported trust situations, trust was experienced more intensely than distrust was experienced in the reported distrust situations.

### Discussion

Study 2 confirmed the importance of several predictors for trust and distrust in IS at work that were observed in Study 1, in particular *Reliability* and *Credibility*. Notably, the significant predictors correspond to the general structure of the IS success model of Delone & McLean (2003). Aspects of system quality, information quality, service quality, and context explained significant amounts of variance in both trust and distrust conditions. However, the findings in Study 1 extended the original IS success model. In particular, person factors such as the perceived ability of persons involved emerged as a new factor that was validated in Study 2. In addition, *Credibility* proved to be an important aspect of information quality in predicting both trust and distrust in Study 2. In the overall analyses of our data, system quality (i.e., *Reliability*) and information quality (i.e., *Credibility*) were revealed as most important.

**Figure 1:**
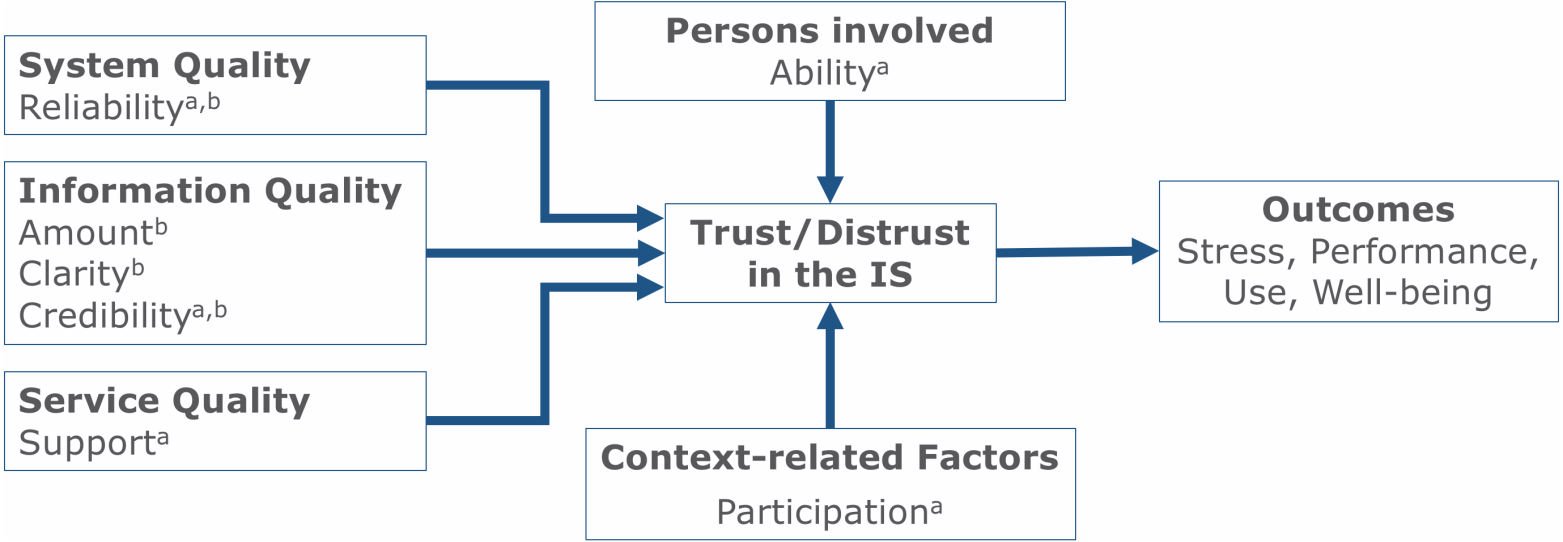
Model of predictors of trust and distrust in IS at the workplace. Variables marked with ^a^ are predictors of trust, variables marked with ^b^ predictors of distrust.

Yet, not all aspects identified in Study 1 were found to be significant predictors of trust or distrust in Study 2. As a consequence, we were able to predict trust and distrust in IS at the workplace using a more parsimonious model than one might have assumed based on Study 1. Figure 1 summarizes our findings and illustrates the different predictors leading to trust or distrust. Variance in the trust situation could be very well explained with *Reliability* and *Credibility* as the two most important predictors of trust. Both predictors were relevant for distrust situations as well, yet less variance was explained, indicating that additional variables might play a role. Notable is the observed suppression effect in the explanation of distrust due to *Clarity* as information quality. *Clarity* seemed to suppress irrelevant variance in *Amount* and *Credibility* of information caused by ambiguities of data in IS.

Comparing the predictors of trust and distrust (see Table 6) showed that many predictors were rated higher for trust, i.e., system and information quality, the context, and persons involved. This is consistent with an overall more positive assessment of trusted as compared to distrusted IS. However, the ratings of distrusted IS were rarely poor—distrust already occurred when IS quality was rated to be mediocre, suggesting that even minor quality problems are enough to turn trust into distrust. IS with major flaws will quite likely lead to even higher distrust in and disuse of the IS.

The importance of trust maintenance is clearly illustrated by the significant and partly high correlations with the considered outcome variables: *Well-being* and *Performance* were rated much higher in trust situations (large effect sizes), *Post-situational satisfaction* with IS use was rated higher in trust situations (small effect size), and experienced stress was rated higher in distrust situations (medium effect size). These results suggest that users’ trust in IS at work can be highly relevant for both the performance and the well-being of the employees using it.

## General Discussion

The research reported in this paper initially explored and validated main predictors for employees’ trust and distrust in IS at work. In a series of two studies, we combined qualitative and quantitative methodologies. Our findings revealed high correlations of trust and distrust with central outcome variables such as users’ reported task performance, stress, and post-situational satisfaction with IS, underlining the strategic importance of considering trust in IS at work. Moreover, the predictors observed were integrated in a new comprehensive model, following a general structure of the IS success model (Delone & McLean, 2003) but also providing various extensions of preexisting models (see Fig. 1).

With respect to main predictors of trust in IS at work, both studies revealed high relevance of technical system quality (in particular *Reliability*) and information quality (in particular *Credibility*). Thus, in both studies, participants stressed that because they heavily depend on IS, these systems consequently must be highly reliable, technically sophisticated, and support work tasks without any malfunctions. *Reliability* has been considered in the more general IS success model, however, not as a predictor of trust but as a facet of system quality (Delone & McLean, 2003). Of course, *Reliability* is a basic and desired quality of any IS, but our research stresses the importance of *Reliability* in work settings: in-depth interviews in Study 1 revealed that employees relied blindly on a well-working IS, but a single major malfunction could have led to absolute distrust and, in consequence, rejection of the IS. The same also applies for *Credibility* as a crucial aspect of information quality. *Credibility* is a vital characteristic of data in an IS, thus it is a specific predictor for resulting trust or distrust. Indeed, credibility has been discussed as an important attribute of Web services and e-commerce (e.g., De Wulf et al., 2006; Metzger, 2007), but our study stressed its high relevance in IS use at the workplace.

Furthermore, in line with prior research on knowledge management systems (e.g., Thatcher et al., 2011), Study 2 found that perceived *Support* is also a central predictor of trust in IS at work. Moreover, *Ability* has been confirmed as person-related predictor of trust in IS. Notably, however, whereas ability is usually related to the trustee (object of trust e.g., Mayer, Davis & Schoorman, 1995; Riegelsberger, Sasse & McCarthy, 2005), in our study ability was related to other involved persons (while system factors reflect the “ability” of the IS as trustee). Thus, users seem to consider not only the power and reliability of the IS as trustee, but also the abilities of those persons involved in the development and maintenance of the IS. In addition, our study underlines the importance of *Participation* for trust in work-related IS, in line with existing research on IS use (e.g., Hartwick & Barki, 1994). Thus, users like to be involved in the acquisition and configuration of IS relevant to their workplaces.

Although trust and distrust with respect to IS at work were predicted by partly the same factors (with different directions), our analyses revealed considerable differences between these two constructs. As stressed in prior research (e.g., Gefen, Benbasat & Pavlou, 2008; Seckler et al., 2015), trust and distrust seem to be somewhat distinct and not located on the same continuum. While trust could be well predicted in our model with a linear combination of five factors, prediction of distrust seemed to be more complex. The existence of a suppression effect could be an indicator for more reflective and evaluative cognitive processes in the formation of distrust. In addition, differences in explained variance indicate that additional factors of distrust were not covered in Study 2 and need to be further investigated. However, both studies revealed clear differences with respect to predictors of trust and distrust in IS at work as compared to trust in Websites and in e-commerce. In work settings, predictors found to be relevant for Web applications such as structural interface design and ease-of-use, security or brand perception (see Seckler et al., 2015) seem to be of no major relevance. These differences might reflect specific differences between everyday work tasks and intrinsically motivated Web surfing or online shopping.

Finally, both of our studies stressed the relevance of performance-related and emotional outcomes of trust in IS at work. Similar to trust in other persons or teams at work (e.g., Breuer, Hüffmeier & Hertel, 2016; Colquitt, Scott & LePine, 2007), trust in IS is not only highly related to performance and use of IS, but it is also related to users’ well-being and stress. Thus, a high quality IS plus sufficient support structures and competent coworkers are not only relevant for enhancing work outcomes, but they are also important for the health and job satisfaction of employees. Our initial findings here might serve as a starting point for future research in these processes.

### Limitations and future research

When interpreting the results of our studies, some limitations should be taken into account, most of which directly point at avenues for future research. First, in light of the scarcity of existing specific research on trust in IS at work, we started with an explorative study examining the viewpoints of professional business users. The applied critical incident technique (Flanagan, 1954) provides in-depth analyses of specific work events and avoids overgeneralization and subjective theories by participants. In Study 2, we used a more systematic quantitative approach but continued our focus on specific work events for similar reasons as in Study 1. A strength of this focus is the multitude of considered work events and IS used, leading to a high external validity of the presented data. Yet, inherent in the method, the self-report data were probably also influenced by individual biases and preferences and are thus not fully comparable to behavioral observations. For instance, users stressed the abilities of other persons involved as a predictor of trust in IS, yet their own reported ability was not related to trust in IS. As a consequence, the current results need to be complemented by less responsive data in future studies, including behavioral observations and external assessments.

Second, even though we were able to find decent effect sizes in Study 2, future research might include larger samples, enabling structural equation modeling to examine the complex interrelations between trust in IS, its antecedents, IS use, and further outcomes. Using structural equation modeling, outcomes such as performance, stress, well-being and use could be modeled simultaneously, considering both performance and users’ well-being as important consequences of trust in IS at work.

Third, we were able to validate relevant predictors for trust in IS at work in Study 2 and examine differences in the formation of distrust. Yet, the explained variance in the regression analyses of distrust was comparably lower than in the regression of trust, indicating that the development of distrust in IS might be more complex and affected by additional variables not investigated in Study 2. Future research is desirable to further explore the differences in IS trust and distrust emergence, as well as in the involved processes at work. The results of more fine-grained research surrounding distrust in IS is particularly important in order to develop interventions to prevent or reverse distrust in IS.

Fourth, all tested participants shared a common cultural background in terms of nationality. Yet, culture plays an important role in the adoption and use of IS (e.g., Jarvenpaa, Cantu & Lim, 2017; Leidner & Kayworth, 2006). Thus, a future cross-cultural investigation and validation of the current findings would be of high value.

### Practical implications

Our results underline that it is imperative to use reliable digital systems at work that provide credible information. Those factors should be central when acquiring and customizing an IS for work tasks. Shortcomings related to these factors could lead to users’ distrust, followed by higher stress and less system use. However, in addition to a reliable system and credible information, managers might also want to ensure sufficient technical support, high abilities of other involved persons, and opportunities to participate in the selection and adoption of the IS in order to enable high levels of trust in the IS. In Table 7, we provide a selection of more detailed practical implications to support trust and to avoid distrust in IS that can be derived from Study 1 and Study 2.

**Table 7:** Practical implications for trust and distrust in IS at the workplace based on findings of Study 1 and Study 2.

How to support trust	How to avoid distrust
*System quality:* - Create situations in which users can have positive experiences with using the IS and that demonstrate its high reliability - Minimize potential errors by implementing controls such as plausibility checks at the level of data entry	*System quality:* - Make sure the IS always works properly without any malfunctions; reliability must be as high as possible
*Information quality:* - Make sure that users notice the high quality of data provided - Incorporate routine checks into the IS, proving the credibility of its data	*Information quality:* - Guarantee the provision of correct data - Ensure that the amount of information provided by the IS is appropriate - Provide data in a clear and concise manner, so that users will be able to detect errors and occasions when an IS rightfully has to be distrusted; offer fast support and correction options for such occasions
*Service quality:* - Arrange that support staff is available in case of problems - Acknowledge the relevance of support staff activities both to users and to the support staff members - Ensure regular maintenance of the IS	
*Context:* - Involve users in decisions/changes concerning the IS - Provide background information on IS functioning	*Context:* - Do not miss opportunities to integrate users in problem solving and developing the IS further
*Persons involved:* - Train users and data management staff regularly - Make transparent to the users how data management staff is trained and how they work	*Persons involved:* - Stress the importance and impact of correct and conscientious use of the IS and data management - Make sure to hire conscientious and competent data managers

From a management point of view, the above-mentioned aspects might seem only of high importance in change processes, when new systems have to be adopted (Leidner & Kayworth, 2006). Yet, in both of our studies participants were highly familiar with the used systems—and still, those factors emerged as important. Thus, the derived predictors of trust and distrust have to be focused on continuously, not only in the implementation phase of an IS. In practice, survey questions in a monitoring study could be based on instruments such as those displayed in Appendix B. In particular, with respect to the most relevant predictors, we can recommend the scale of McKnight et al. (2011) to investigate *Reliability*. For measuring *Credibility*, we suggest the corresponding scale of the Web-CLIC (Thielsch & Hirschfeld, in press) or an adoption of the message credibility scale of Appelman & Sundar (2016). Such scales are short enough to be enclosed in employee surveys or other monitoring studies targeted at the IT infrastructure of an organization.

### Conclusions

The digitalization of work and growing presence of complex IS in many work organizations requires a sound understanding of users’ trust in these systems. The present research provides one of the first empirical explorations and validations of key predictors for trust and distrust in IS at the workplace. *Reliability, Credibility, Support, Ability of involved persons*, and *Participation* of employees were revealed as crucial for the prediction of trust. However, distrust was partly predicted by different factors, reflecting different cognitive and evaluative processes. In general, whereas trust is maintained via additive combination of several factors (see Table 7), distrust can be triggered by a single problem, even a relatively minor one. Based on the reported results, our comprehensible model of trust in IS at work (see Fig. 1) provides a more thorough understanding of as well as practical guidance for successful adoption, diffusion, and use of IS in work settings, particularly in situations where workers feel vulnerable due to uncertain and risky work conditions (Wang & Emurian, 2005). Such feelings result not only from work tasks, but they can also be caused by time pressure, high potential gains or losses, and a high degree of personal responsibility; factors that are quite typical for many work processes today.

##  Supplemental Information

10.7717/peerj.5483/supp-1 Supplemental Information 1 Appendix

10.7717/peerj.5483/supp-2 Supplemental Information 2 Data package for study 2 Data package contains raw data for study 2 of “Trust and Distrust in Information Systems at the Workplace”. Demographic variables were excluded to protect privacy of participants. (Study 1 was an interview study using the critical incidents technique. Thus, data of study 1 (i.e., audio files) were not provided to protect privacy of the interviewees).

## Additional Information and Declarations

### Data Availability

The following information was supplied regarding data availability: The raw data are provided in the Supplemental Files.
