# Supplementary material for: Trust and distrust in information systems at the workplace
Source: PeerJ. 2018 Sep 12;6:e5483. doi: 10.7717/peerj.5483 (PMC6139009; doi:10.7717/peerj.5483)
Supplement: Supplemental Information 1 [file peerj-06-5483-s001.pdf]

Appendix A

Interview Guide for Study 1

1. Critical incident for trust:

Instructions

"Please remember a situation, in which you trusted an information system. In this situation, you should have relied heavily on the information system and you should have carried out a concrete action with the information system. Please describe all of the circumstances, and above all, please describe which prevailing conditions caused your trust in this situation."

Further questions asked if necessary:

- Exactly what contributed to the fact that you did rely on the information system?
- Which action did you carry out in this situation?
- How did you react in this situation?
- How did you feel in/perceive this situation?
- How did this situation affect your further use of the system?
- What were the consequences of that situation?
- Where there other people involved in this situation? Who?
- When/how often did this situation occur?
- Were there further aspects that were important in this situation?

2. Critical incident for distrust:

Instructions

"Please remember a situation, in which you distrusted an information system. In this situation, you should have relied very little on the information system and you should have omitted a concrete action with the information system. Please describe all of the circumstances, and above all, please describe which prevailing conditions caused your distrust in this situation."

Further questions asked if necessary:

- Exactly what contributed to the fact that you did not rely on the information system?
- Which action did you carry out in this situation?
- How did you react in this situation?
- How did you feel in/perceive this situation?
- How did this situation affect your further use of the system?
- What were the consequences of that situation?
- Where there other people involved in this situation? Who?
- When/how often did this situation occur?
- Were there further aspects that were important in this situation?

3. Description of the information system and the use of it regarding:
   - Tasks underdone with the system
   - (Daily) utilization time
   - Other users
   - Potential alternative systems
   - Implementation process
   - Training
   - General evaluation of user experience

4. Conclusion:
   - Do you want to add any further information?

Appendix B

Full Item List for Study 2

Person related measures

Technology competence (Neyer et al., 2012; α = .84)

1. Dealing with modern technology, I am often afraid of failing.
2. For me, dealing with technological innovations mostly represents an excessive demand.
3. I am afraid that I rather destroy technological innovations than that I use them properly.
4. I find it hard to deal with new technology – mostly I am just not able to do so.

Disposition to trust technology (McKnight et al., 2011; α = .86)

1. I usually trust a technology until it gives me a reason not to trust it.
2. I generally give a technology the benefit of the doubt when I first use it.
3. My typical approach is to trust new technologies until they prove to me that I shouldn't trust them.

[Recall of the critical incident]

Information about the system and its use

1. What is the name of the information system that you use? (not cumpolsory)
2. Since when have you already been working with the information system? (*years/months*)

Regular Frequency of use **(person related measure)**

1. How often do you use the information system actively, i.e. carrying out a concrete action? (*never/once a year/several times a year/once a month/several times a month/once a week/several times a week/on a daily basis*)

Obligation to use the IS (Moore & Benbasat, 1991; α = .82)

1. I am expected to use the information system.
2. Using the information system is compulsory in my job.

Information on the IS

1. Which kind of data are mainly administered with the system? (*patient data/customer data/HR data/product data/business data/programming data/measurement data/others*)
2. When did the situation occur? (*more than a year ago/during the last year/during the last month/during the last week/today*)
3. How often did the situation occur? (*once/once a year/several times a year/ once a month/several times a month/once a week/several times a week/on a daily basis*)
4. Which of the following categories describes the situation best? (*data retrieval/data input & management/data security/automated processes/system implementation & support/other*)

Situational Trust/Distrust

1. I completely trusted/distrusted the information system.
2. I heavily/not at all relied on the information system.
3. I felt comfortable/uncomfortable relying on the information system.

System Quality

Reliability (McKnight et al., 2011; four item scale: $\alpha = .90$)

1. The information system is a very reliable piece of software.
2. The information system is extremely dependable.
3. The information system does not malfunction for me.

Implemented controls (Rivard et al., 1997; five item scale: $\alpha = .73$)

1. The system does not destroy any information without asking for a confirmation and getting a positive response.
2. The application provides default values at the data-entry level.
3. Recovery and retrieval procedures are available in case of an application malfunction.

Ease of use (Gefen & Keil, 1998; $\alpha = .89$)

1. Learning to operate the system was easy for me.
2. Using the system is clear and understandable.
3. I believe that the system is easy to use.

Response Time (Palmer, 2002; $\alpha = .70$)

1. The rate at which the system provided information was fast enough.
2. The rate at which the information was displayed was fast enough.

Information quality

Amount (Lee et al., 2002; four item scale: $\alpha = .76$)

1. The information is of sufficient volume for my needs.
2. The amount of information is neither too much or too little.

Relevance (Lee et al., 2002; four item scale: $\alpha = .94$)

1. The information is useful to my work.
2. The information is relevant to my work.
3. The information is applicable to my work.

Security (Lee et al., 2002; four item scale: $\alpha = .81$)

1. The information is protected against unauthorized access.
2. Access to this information is sufficiently restricted.
3. The information can only be accessed by people who should see it.

Informativeness (Thielsch & Hirschfeld, in press; $\alpha = .87$-91)

1. The information provided by the system is of high quality.
2. I find the information to be useful.
3. The system is informative.

Credibility (Thielsch & Hirschfeld, in press; $\alpha = .93$-95)

1. I find the information provided to be authentic.
2. I can trust the information.
3. The information provided is reliable.

Clarity (Thielsch & Hirschfeld, in press; $\alpha = .83$)

1. The contents are clearly presented.
2. The language in the system is current and easy to understand.
3. The system provide me information in a clear and concise manner.

Service Quality

Support

1. If problems occur, a support is available.
2. The system is maintained regularly.
3. The support is helpful.

Context related measures

Participation (Baroudi & Orlikowski, 1988; $\alpha = .89$)

1. I am sufficiently provided with information about changes and decisions concerning the system.
2. I am sufficiently involved in changes and decisions concerning the system.

Transparency (Baroudi & Orlikowski, 1988; $\alpha = .88$)

1. I have sufficient background information about the system.
2. I have sufficient information about the operating principles of the system.

Error communication (Cigularov et al., 2010; five item scale: $\alpha = .89$)

1. When people are unable to correct an error by themselves, they turn to their co-workers
2. When people make an error, they can ask others for advice on how to continue.
3. If people are unable to continue their work after an error, they can rely on others.

Perceived Organizational support (Eisenberger et al, 2001; six item scale: $\alpha = .77$)

My employer

1. …really cares about my well-being
2. …strongly considers my goals and values.
3. …is willing to help me if I need help.

Persons involved

Abilities (Hertel at al., 2004)

1. The qualification of persons involved is sufficient.
2. I trust in the professional competence of persons involved.
3. I think I can rely on the skills of persons involved.

Attitudes (Hertel at al., 2004)

1. I trust in the motivation of persons involved.
2. The commitment of persons involved is sufficient.
3. I think persons involved have high standards for performing their tasks.

Accountability (Frink & Ferris, 1998; $\alpha = .91$)

1. I feel accountable for my work to my team members.
2. I feel accountable for my work to my superiors.
3. I feel accountable for my work to clients/customers/patients.

Consequences

Well-being (Jäger, 2004)

1. *Smiley rating scale*

Stress (Stanton, Balzer, Smith, Parra, & Ironson, 2001)

1. How demanding was the situation for you?
2. How stressed did you feel in the situation?
3. How onerous was the situation for you?

Performance (Etezadi & Farhoomad, 1996; $\alpha = .91$)
In the situation, how successful has the system been

1. …in improving the quality of your work?
2. …in making your job easier?
3. …in saving you time?
4. …in helping you fulfil the needs and requirements of your job?

Post-situational frequency of use

1. After the situation, my use of the system… (*strongly decreased/decreased/rather decreased /neutral/rather increased/increased/strongly increased*)

Post-situational satisfaction with use

1. After the situation, I enjoyed using the system.

In addition to the variables suggested by the results of Study 1 and prominent within the trust literature, we explored three further user variables (Need for cognition, Need for control, Conscientiousness) and two system quality variables that are discussed in the e-commerce literature (Aesthetics and Customizability). Yet, none of these showed any meaningful relation in Study 2 and including them did not change the present results. Data are enclosed in the data file (see supplement), items are as follows:

User variables
Need for cognition (Beissert, Köhler, Rempel, & Beierlein, 2014; $\alpha = .51\text{-}54$)

1. Simply knowing the answer rather than understanding the reasons for the answer to a problem is fine with me.
2. I prefer my life to be filled with puzzles that I solve.
3. I would prefer complex to simple problems.
4. I primarily think because I have to.

Need for control (De Rijk, Blanc, Schaufeli, & de Jonge, J., 1998)
In my job, I set a great store by,

1. …being able to set the pace of my tasks.
2. …having control over what I do and the way I do it.
3. …doing my own planning.

Conscientiousness (Schupp & Gerlitz, 2014; $\alpha = .72$)
I see myself as someone who

1. … does a thorough job.
2. … tends to be lazy.
3. … does everything efficiently.

System Quality
Aesthetics

1. The design of the system is appealing.

Customizability

1. I can adjust the system according to my needs.
2. The system is customizable.
