# Supplementary material for: Trust and distrust in information systems at the workplace
Source: PeerJ. 2018 Sep 12;6:e5483. doi: 10.7717/peerj.5483 (PMC6139009; doi:10.7717/peerj.5483)
Supplement: Supplemental Information 2 — Data package contains raw data for study 2 of “Trust and Distrust in Information Systems at the Workplace”. Demographic variables were excluded to protect privacy of participants. (Study 1 was an interview study using the critical incidents technique. Thus, data of study 1 (i.e., audio files) were not provided to protect privacy of the interviewees). [file peerj-06-5483-s002.zip › Data_package_study2/Study 2_Notes for Analysis.pdf]

Notes for analysis

Trust and Distrust in Information Systems at the Workplace

Study 2

Raw data table: raw data_Study 2.csv

Note: Items with a linguistically negative formulation (e.g., “In dealing with modern computer technologies I am afraid of failing.”): TKomp1, TKomp2, TKomp3, TKomp4, NfCog1, NfCog4, Gew2. These items’ **scales need to be recoded** so that higher scores for the item represent higher scores for the relating construct.

Constructs and depiction in raw data table

Item/Variable	Construct	Scale
lfdn	Serial number	
C_0001	Incident condition	0=Trust, 1=Distrust
ZustTeiln	Consent to conditions of paricipation	1 = ja / yes; 2 = nein / no
TKomp1-TKomp4	Technology Competence	1 = stimme gar nicht zu / strongly disagree, 2 = stimme nicht zu / disagree, 3 = stimme eher nicht zu / rather disagree, 4 = weder noch /neither, 5 = stimme eher zu / rather agree, 6 = stimme zu / agree, 7 = stimme voll zu / strongly agree
ViT1-ViT3	Trust in Technology	``
NfCog1-NfCog4	Need for Cognition	``
Gew1-Gew3	Conscientiousness	``
NfCont1-NfCont3	Need for Control	``
noSit	no situation remembered	1=Ich erinnere keine Situation / I don’t remember a situation, 0=Situation erinnert / situation remembered
noSit2	really no situation remembered; appeared if no Sit was answered with 1= I don’t remember a situation	1= Ich erinnere eine Situation / I remember a situation, 2= Ich erinnere keine Situation (Befragung beenden) / I **don’t** remember a situation (exit survey); -77= Seite nicht erreicht / page was not reached
NameIS	Name of the IS	Freitext / free entry
NJahre	Time of use in years	Jahre / years
NMonate	Time of use in month	Monate / month
N	Total time of use (calculated from NJahr and NMonate)	Jahre / years
NHaeufigk	Nutzungshäufigkeit	1=Nie / never, 2=ca.1x pro Jahr / app. Onec a year, 3=mehrmals pro Jahr / several times a year, 4=ca. 1x Pro Monat / app. Once a month, 5= Mehrmals pro Monat / several times a month, 6= ca. 1x pro Woche / once a week,

		7=mehrmals pro Woche / several times a week
Obl1-Obl2	Obligation to use	1 = stimme gar nicht zu / strongly disagree, 2 = stimme nicht zu / disagree, 3 = stimme eher nicht zu / rather disagree, 4 = weder noch /neither, 5 = stimme eher zu / rather agree, 6 = stimme zu / agree, 7 = stimme voll zu / strongly agree
Datentyp	Type of data administered with the IS	1=Patientendaten (z.B. Kranken- und Pflegemanagement-Systeme) / patient data (e.g., health or nursing management systems), 2=Kundendaten (z.B. Kundenmanagement/Customer Relationship-Systeme) / customer data (e.g., customer management/customer releationship systems), 3=Personaldaten (z.B. Personal-/HR-Software) / employee data (e.g., personnel/HR systems) , 4=Produktdaten (z.B. Warenwirtschafts-/Enterprise Resource Planning-Systeme) / product data (product management/ERP systems), 5=Geschäftsdaten (z.B. Business Intelligence System, Reporting Tools) / business data (e.g., business intelligence systems, reporting tools), 6=Programmierungsdaten (z.B. Versionskontrollsysteme) / programming data (e.g., version control systems), 7= Vermessungsdaten (z.B. Katastersysteme) / measurement data (e.g., cadastral systems), 8=Sonstiges (Freitext) / others (free entry)
SitHaeufigk	Frequency of the incident’s occurrence	1=Einmalig / once, 2=ca. 1x pro Jahr / app. once a year, 3=mehrmals pro Jahr / several times a year, 4=ca. 1x pro Monat / app. Once a month, 5=mehrmals pro Monat / several times a month, 6=ca. 1x pro Woche / app. Once a week, 7=mehrmals pro Woche / several times a week, 8=täglich / everyday

SitZeitp	Date of the incident's last occurrence	1=Vor mehr als einem Jahr / more than one year ago, 2=Innerhalb des letzten Jahres / within the last year, 3=Innerhalb des letzten Monats / within the last month, 4=Innerhalb der letzten Woche / within the last week, 5=Heute / today
SitKat	Type of situation<br>* „alles oben genannte, besonders Ausfallsicherheit und Datenschutz [all oft he above mentioned, above all failure savety and data protection]" was coded as = 3; „Arbeitszeitüberprüfung (Salden), Krankheit (BEM) [audit of working time and sick days]", „Kalkulationen [calculations]", and „Vorschlag zur Neudimensionierung eines Lagers [Proposal for rescaling of a warehouse]", „Berechnungen [calculations]", „Auswertung von Daten [data analysis]" were coded as = 1	1=Informationsabfrage / information retrieval, 2=Dateneingabe & -verwaltung / data entry & administration, 3=Datenschutz & Sicherheit / data protection & security, 4=Automatisierung (automatische Vorgänge) / automation (automated processes), 5=System- bzw. Funktionseinführung & Support / implementation of system or functions & support, 6=Sonstiges, und zwar [Textfeld für offene Antwort] / others, namely [free entry]*
SitVert1-SitVert3	Situational trust or distrust (in accordance with c_0001)	1 = stimme gar nicht zu / strongly disagree, 2 = stimme nicht zu / disagree, 3 = stimme eher nicht zu / rather disagree, 4 = weder noch /neither, 5 = stimme eher zu / rather agree, 6 = stimme zu / agree, 7 = stimme voll zu / strongly agree
Rel1-Rel3	Reliability	``
Contr1-Contr3	Implemented Controls	``
EOU1-EOU3	Ease of Use	``
Custom1-Custom2	Customizability	``
Aestet	Aesthetics	``
InfoMenge1-InfoMenge2	Amount of Information	``
InfoRelev1-InfoRelev3	Relevance of Information	``
Sec1-Sec3	Perceived Security	``
Informative1-Informative3	Informativeness of Information	``
Cred1-Cred3	Credibility of Information	``
Clar1-Clar3	Clarity of Information	``
Resp1-Resp2	Response Time	``
Partip1-Partip2	Participation	``
Transp1-Transp2	Transparency	``
Support1-Support3	Support	``
bPAbility1- bPAbility3	Abilities of persons involved	1 = stimme gar nicht zu / strongly disagree, 2 = stimme nicht zu / disagree, 3 = stimme eher nicht zu / rather disagree, 4 = weder noch /neither, 5 = stimme eher zu / rather agree, 6

		= stimme zu / agree, 7 = stimme voll zu / strongly agree, NA = trifft nicht zu / not applicable
bPAttitude1- bPAttitude3	Attitude of persons involved	``
bPAccount1- bPAccount3	Accountability	``
ErrCom1-ErrCom3	Error Communication	1 = stimme gar nicht zu / strongly disagree, 2 = stimme nicht zu / disagree, 3 = stimme eher nicht zu / rather disagree, 4 = weder noch /neither, 5 = stimme eher zu / rather agree, 6 = stimme zu / agree, 7 = stimme voll zu / strongly agree
POS1-POS3	Perceived Organizational Support	``
Smiley	Well-Being	``
Stress1-Stress3	Stress	1=Sehr wenig / very little, 2=wenig / little, 3=eher wenig / rather little, 4=unentschieden /undecided, 5=eher stark / rather strongly, 6=stark / strongly, 7=sehr stark / very strongly
Perform1-Perform4	Performance	1=gar nicht erfolgreich / not at all successful, 2=nicht erfolgreich / not sucessful, 3=eher nicht erfolgreich / rather not successful, 4=weder noch / neither, 5=eher erfolgreich / rather successful, 6=erfolgreich / sucessful, 7=voll erfolgreich / very sucessful
EffektHaeufigk	Use after the incident	1=stark abgenommen / strongly decreased, 2=abgenommen / decreased, 3=eher abgenommen / rather decreased, 4=weder noch / neither, 5=eher zugenommen / rather increased, 6=zugenommen / increased, 7=stark zugenommen / strongly increased
EffektEinstN	Satisfaction with use after the incident	1 = stimme gar nicht zu / strongly disagree, 2 = stimme nicht zu / disagree, 3 = stimme eher nicht zu / rather disagree, 4 = weder noch /neither, 5 = stimme eher zu / rather agree, 6 = stimme zu / agree, 7 = stimme voll zu / strongly agree
SA	Self-exclusion	1=Daten dürfen verwendet werden / data may be used, 2=Ausschluss / exclusion
